# Microstructure Evolution and a Unified Constitutive Model of Ti-55511 Alloy Compressed at Stepped Strain Rates

**DOI:** 10.3390/ma14226750

**Published:** 2021-11-09

**Authors:** Gang Su, Zhong Yun, Yong-Cheng Lin, Dao-Guang He, Song Zhang, Zi-Jian Chen

**Affiliations:** 1 Light Alloy Research Institute, Central South University, Changsha 410083, China; sugang@csu.edu.cn (G.S.); 203801010@csu.edu.cn (S.Z.); zjchen@csu.edu.cn (Z.-J.C.); 2 School of Mechanical and Electrical Engineering, Central South University, Changsha 410083, China; daoguanghe@csu.edu.cn; 3 State Key Laboratory of High Performance Complex Manufacturing, Changsha 410083, China

**Keywords:** titanium alloy, flow behavior, constitutive model, microstructure, softening mechanisms

## Abstract

The flow behavior and microstructure change of the Ti-55511 alloy are investigated by thermal compression experiments with stepped strain rates. The phase transformation features, the dynamic recrystallization (DRX) behavior of the β matrix, the dynamic spheroidization mechanism of the lamellar α phase and the evolution of the β sub-grain size are quantitatively analyzed. A unified constitutive model is constructed to characterize the hot deformation features of the Ti-55511 alloy. In the established model, the work hardening effect is taken into account by involving the coupled effects of the equiaxed and lamellar α phases, as well as β substructures. The dynamic softening mechanisms including the dynamic recovery (DRV), DRX and dynamic spheroidization mechanisms are also considered. The material parameters are optimized by the multi-objective algorithm in the MATLAB toolbox. The consistency between the predicted and experimental data indicates that the developed unified model can accurately describe the flow features and microstructure evolution of the hot compressed Ti-55511 at stepped strain rates.

## 1. Introduction

Due to the advantages of excellent strength and high elastic modulus/fracture toughness/hardness, the near β titanium alloys are widely used in some key aviation components such as aircraft landing gear, wing connection fasteners, etc. [1,2,3]. As a typical near β titanium alloy, the Ti-5Al-5Mo-5V-1Cr-1Fe (Ti-55511) alloy with a good balance of strength and breaking tenacity is widely applied in manufacturing the important aviation components. Generally, the mechanical capacities of the β titanium alloy are effectively improved by the thermomechanical process [4,5]. In recent years, the thermomechanical process of Ti-55511 alloy has been investigated by isothermal compression experiments, in which the deformation parameters (temperature, strain rate) are constant [6,7,8]. However, the processing parameters are time-variant in the practical forging process of parts. Therefore, it is significant to investigate the hot deformation behavior and microstructure evolution of the Ti-55511 alloy by dynamic compression experiments with time-varying strain rates.

During the isothermal compression, the alloy undergoes complex deformation mechanisms such as work hardening (WH), dynamic recovery (DRV) and dynamic recrystallization (DRX), etc. [9,10,11,12], which often influence flow stress and microstructures. A unified constitutive model is one ideal method to describe the relationship between flow behavior, deformation parameters and microstructure evolution. Up to now, some researchers have concentrated on the constitutive models for different metals or alloys such as aluminum alloys [13], superalloys [14,15,16], titanium alloys [4,5,17,18,19,20], steels [21,22,23], etc. In general, these models can be divided into three types, namely phenomenological models, machine learning models and physical-based models. The Arrhenius-type equation is one of the most widely used phenomenological models [24]. Gastro et al. [25] used the genetic algorithm (GAd) to optimize the material constants in the strain-compensated Arrhenius equation. Lin et al. [26] established the Arrhenius-type phenomenological, artificial neural network (ANN) and multi-gene genetic programming (MGGP) models to predict the flow stress of a Ni-based superalloy. Quan et al. [27] predicted the true stress and phase transformation behavior of the hot compressed as-cast Ti-6Al-2Zr-1Mo-1V by a back-propagation neural network (BPNN). Lin et al. [17] validated that the BPNN model is more suitable than the Hensel–Spittel (HS) model and the strain-compensated Arrhenius equation for describing the hot defamation behavior of the Ti-55511 alloy. He et al. [13] established an accurate e-insensitive support vector regression (e-SVR) model to predict the flow stress of the hot compressed GH4169 alloy (a Ni-based superalloy). However, the phenomenological models and artificial intelligence models cannot directly reflect the relationship between the material deformation physical mechanisms, microstructure change and flow stress. Therefore, the physical-based internal state variable (ISV) models considering the complicated deformation mechanisms have been popular over the past decades [28]. The WH and DRV processes were modeled in early research [29,30], and the dislocation density was introduced as the main ISV in the Kocks–Meching (K–M) model. With the development of physical-based ISV models, some other complicated deformation mechanisms were also considered, such as DRX [14,31,32,33], spheroidization [15,34,35] and precipitation [11], which can greatly influence the flow stress. Recently, many investigations have been made to introduce these physical mechanisms into constitutive models. Liang et al. [10] identified the relationship between the critical dislocation density for DRX and the saturated dislocation density of the Ti-55511 alloy. Tang et al. [11] constructed a physical-based ISV model, in which the precipitation, static recovery/recrystallization and precipitation were coupled, to predict flow stress and microstructure during the multi-stage hot compression of the Al-Zn-Mg-Cu alloy. Tang et al. [14] and Lin et al. [25] established a unified ISV model considering the DRX fraction and average grain size to predict the flow stress and microstructure change of Ni-based superalloys. Xiao et al. [15] developed a dislocation-based model to predict the flow stress and spheroidization behavior of the Ti-55511 alloy. Peng et al. [28] proposed a physical-based model to predict the flow behavior of the TA15 alloy with a lamellar colony α phase. Li et al. [29] developed a set of ISV unified viscous-plastic equations to model the flow stress and spheroidization behavior of a TC6 alloy. Wu et al. [30] modeled the flow behavior and microstructure change of a hot compressed Ti2AlNb via a set of ISV-based unified equations, which coupled the dislocation density, DRX, subgrains size, phase volume fraction, spheroidization, damage evolution and deformation heating.

Still, the relationships between the flow behavior, deformation mechanisms and microstructure evolution of the Ti-55511 alloy at time-varying strain rates are not clear. Therefore, this work is aimed at developing a unified physical-based model to forecast the flow stress, as well as revealing the relationship between the flow behavior and intrinsic deformation mechanisms, of a Ti-55511 alloy at time-varying hot deformation parameters. There are the following several parts. Firstly, the flow behavior and microstructure evolution of a Ti-55511 alloy compressed at stepped strain rates are investigated. Secondly, based on the measured flow stress and deformation mechanisms, a unified model incorporating dislocation density, phase transformation, spheroidization of α phase and DRX of β grains is developed. Finally, the accuracy of the developed model is verified. This work can provide the theoretical guidance for the isothermal forging process.

## 2. Materials and Experimentations

### 2.1. Materials

**Figure 1:**
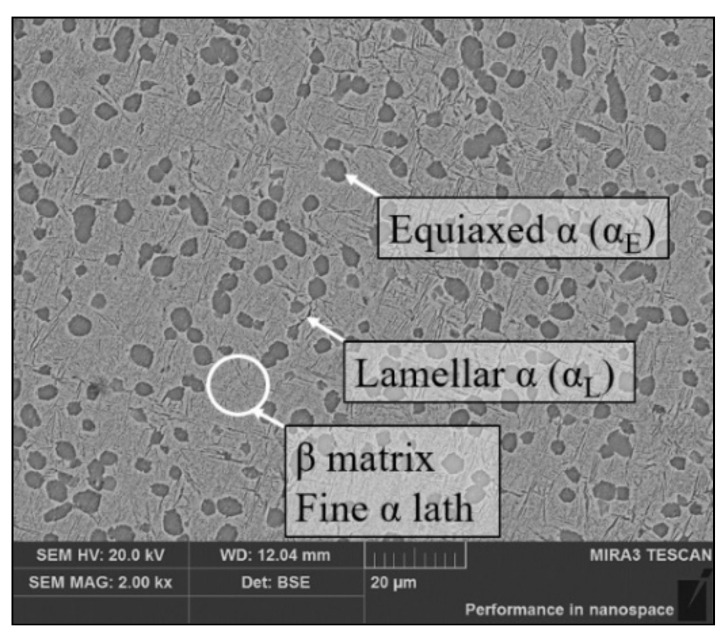
The initial microstructure of the Ti-55511 alloy.

A forged Ti-55511 alloy with chemical compositions (wt.%) of 5.16Al-4.92Mo-1.10Cr-0.98Fe-Ti (bal.) was used. The α/β phase transition temperature was 1148K [17]. Figure 1 shows the initial microstructure of the Ti-55511 alloy observed by scanning electron microscope (SEM) (FEI Electron Optics B.V; Prague, Czech Republic). In Figure 1, there are some thick lamellar α phases (α_L_) at the boundaries between subgrains/grains of the β matrix. Such behavior is related to the so-called complete and incomplete wetting of grain boundaries by the second solid phase in the β→α transformation process [36,37], but those continuous grain boundary α_L_ are deleterious to the material ductility [38]. In the subsequent hot deformation process, those α_L_ can be spheroidized and the material ductility can be improved.

The volume fractions of the equiaxed α phase (α_E_), α_L_ and β phase were counted as 25.44%, 14.47% and 60.9%, respectively, by Image-Pro Plus 6.0 software. The average diameter of the α_E_ phase was 3.1 μm and the average thickness of the α_L_ phases was 0.245 μm. In addition, many fine lamellar α phases were dispersed in the β matrix.

### 2.2. Hot Compression Test with Stepped Strain Rate

The hot compression experiments with stepped strain rates were performed on a Gleeble-3500 simulator under isothermal conditions. The size of specimens was Φ10 mm × 15 mm. The K-type thermocouple was used to measure the temperature of specimens. The detailed experiment procedure is illustrated in Table 1.

Before the deformation, the specimens were first heated to the preset temperatures (973–1063 K) at 10 °C/s, holding for 300 s to obtain the uniform temperature field in specimens. Secondly, the specimens were compressed to the true strain of stage I ($\varepsilon_{I}$) with the strain rate of stage I (${\dot{\varepsilon }}_{1}$). Then, the strain rate was immediately changed to the strain rate of stage II (${\dot{\varepsilon }}_{2}$) and the further deformation was continued until the total strain (ε) of 0.92. Finally, the water quenching was carried out to preserve the deformed microstructure. Here, the value of ${\dot{\varepsilon }}_{1}$ was 0.1 s^−1^. The ranges of $\varepsilon_{I}$ and ${\dot{\varepsilon }}_{2}$ were 0.2–0.5 and 0.001–1 s^−1^, respectively.

### 2.3. Microstrucuture Observation

The microstructures in the compressed specimens were observed by SEM (FEI Electron Optics B.V; Prague, Czech Republic), electron backscatter diffraction (EBSD) (FEI Electron Optics B.V; Prague, Czech Republic) and transmission Electron Microscopy (TEM) (Tecnai G2 F20; FEI company; Hillsboro, OR, USA). All specimens were split along the compression axis and metallographically prepared. For SEM observation, the etching solution of HNO_3_ (2 mL) +HF (6 mL) + H_2_O (92 mL) was used to etching specimens, and Image-Pro Plus 6.0 was used to calculate the fraction of phases. For EBSD and TEM observations, the foils were ground to about 80 μm, then electropolished by a twin-jet electropolisher (5% perchloric + 35%normal butanol + 60% methanol, −25 °C and the voltage of 25 V). The EBSD data were analyzed by the MATLAB toolbox METX5.6.0. The sampling step size was 0.2 μm. The detailed preparation process of samples for EBSD and TEM observations was consistent with those reports in the previous work [1,6].

## 3. Flow Behavior and Microstructure Evolution

### 3.1. Flow Characteristics Deformation Mechanisms

**Figure 2:**
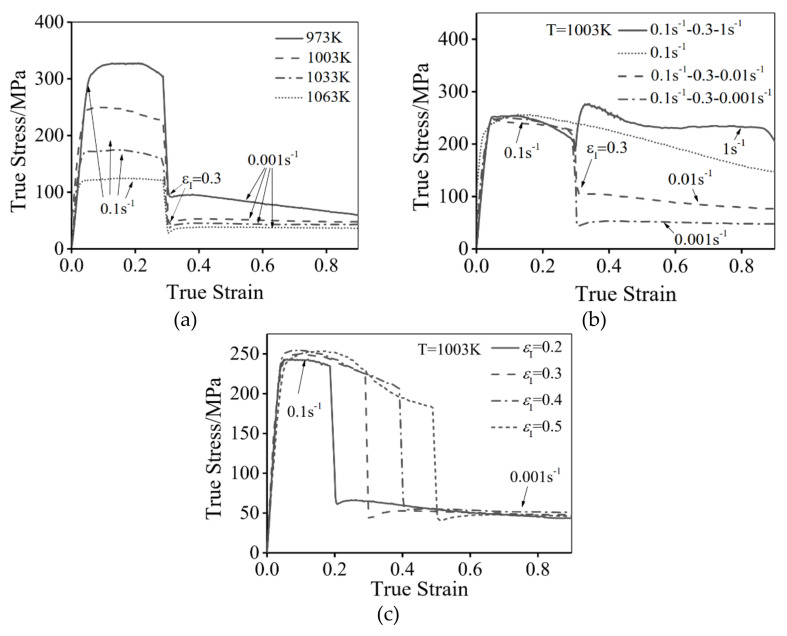
Flow stress curves of Ti-55511 alloy compressed at different: (**a**) *T*; (**b**) ${\dot{\varepsilon }}_{2}$; (**c**) $\varepsilon_{I}$.

Figure 2 presents the typical true stress-strain curves of the hot compressed Ti-55511 alloy at stepped strain rates and different temperatures.

As can be seen, the flow stress is sensitive to the deformation temperature (*T*), stepped strain rate and $\varepsilon_{I}$. The results show that the peak stress appears in all flow stress curves, indicating the deformation process involves the hardening and softening mechanisms, which are caused by the dislocations stacking and annihilation [14]. In particular, when the strain rate is suddenly decreased at $\varepsilon_{I}$, the flow stress quickly decreases. This is because the deformation at a high ${\dot{\varepsilon }}_{1}$ induces many dislocations, which promotes the DRX nucleation-growth behavior in stage II. Meanwhile, many dislocations are consumed in this process. In addition, the studied alloy is sensitive to the strain rate. The lowered strain rate rapidly decreases the increasing rate of dislocations density in stage II. So the flow stress decreases at the beginning of stage II [39]. On the contrary, when the strain rate is suddenly increased, the opposite results are obtained. In addition, the flow stress decreases with an increased T or a decreased ${\dot{\varepsilon }}_{2}$, as shown in Figure 2. This is because the dislocation motion can be enhanced at high temperatures [40]. and the low strain rate can provide sufficient time for the dislocations’ annihilation [15,41].

### 3.2. Microstructure Evolution

#### 3.2.1. Phase Transformation Characteristics

**Figure 3:**
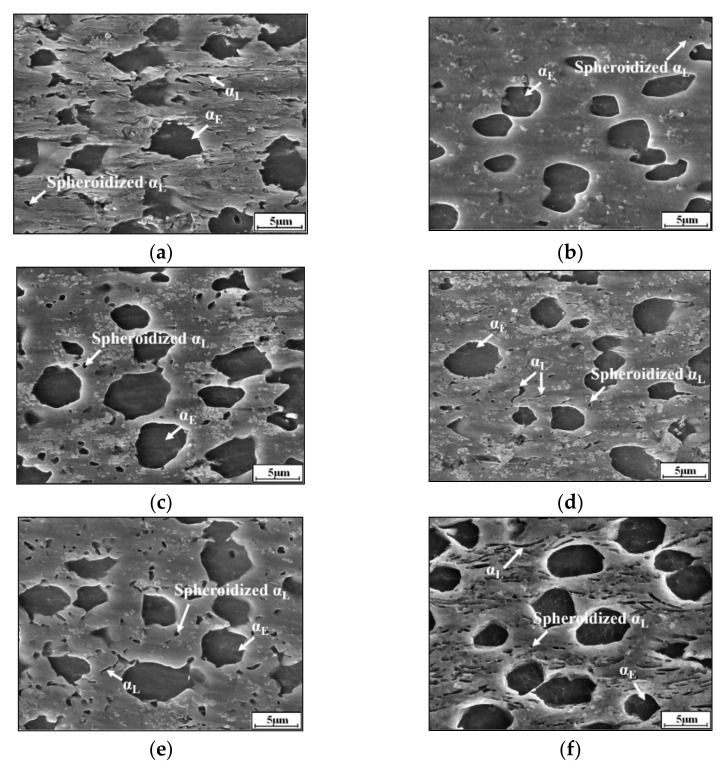
SEM images of Ti-55511 compressed at: (**a**) 973 K–0.1 s^−1^–0.3–0.001 s^−1^; (**b**) 1033 K–0.1 s^−1^–0.3–0.001 s^−1^; (**c**) 1003 K–0.1 s^−1^–0.2–0.001 s^−1^; (**d**) 1003 K–0.1 s^−1^–0.5–0.001 s^−1^; (**e**) 1003 K–0.1 s^−1^–0.3–0.01 s^−1^; (**f**) 1003 K–0.1 s^−1^–0.3–1 s^−1^.

Phase transformation plays a vital role in the mechanical properties of two-phase titanium alloys [20]. Figure 3 shows the phase transformation features under different deformation conditions. The content of β phase increases from 69.99% to 87.73% with an increased temperature from 973 K to 1033 K, as shown in Figure 3a,b.

This indicates that the high temperature can accelerate the phase transformation process from α phase to β matrix [20]. Comparing Figure 3c,d, the fraction of β phase at 1003 K–0.1 s^−1^–0.2–0.001 s^−1^ is higher than that at 1003 K–0.1 s^−1^–0.5–0.001 s^−1^, i.e., the increase of $\varepsilon_{I}$ can restrain the transition of α phase to β matrix. This is because that the larger $\varepsilon_{I}$ (with 0.1 s^−1^) decreases the time for the transformation of α→β in stage II (with 0.001 s^−1^). Similarly, when deformation temperature is 1003 K and $\varepsilon_{I}$ is 0.3, the fraction of β phase decreases obviously with increasing ${\dot{\varepsilon }}_{2}$ (Figure 3e,f). So, the contents of α and β phases are effectively influenced by deformation parameters.

#### 3.2.2. Phase Transformation Characteristics

Figure 3 shows the microstructure of Ti-55511 alloy deformed under different conditions. Generally, the dynamic spheroidization is the main characteristic of lamellar α phases during hot deformation [1,6]. In order to analyze the spheroidization behavior of lamellar α phases, the volume fractions of α_L_ phase (length-width ratio >3), α_E_ phase (length-width ratio 1–3 and average size >1), spheroidized α_L_ phase (α_SP_, length-width ratio 1–3 and average size <1) [6] and β phases are counted quantitatively, as shown in Table 2. The spheroidization fraction (S) is defined as

(1) $$S=\frac{A_{spheroidized}}{A_{lamellar}}$$

where $A_{spheroidized}$ is the area of α_SP_ phase, $A_{lamellar}$ is the area of all α_L_ phases (including spheroidized and incomplete spheroidized α phases).

In Figure 3a, the microstructures consist of α_L_, α_E_ and α_SP_ phases at 973 K. The volume fractions of α_L_, α_E_ and α_SP_ phases are 4.14%, 24.4% and 1.47%, respectively. The corresponding value of S is 26.15%. When the temperature is 1033 K (Figure 3b), most of the α_L_ phase spheroidizes and the value of S is 91.75%. This is because the high temperature promotes the dislocation motion and element diffusion, accelerating the spheroidization process [1,6]. In addition, the α_L_ phase can completely spheroidize at 1003 K–0.1 s^−1^–0.2–0.001 s^−1^ (Figure 3c), while the incomplete spheroidization of the α_L_ phase is dominant at 1003 K–0.1 s^−1^–0.5–0.001 s^−1^ (Figure 3d). This is because the high $\varepsilon_{I}$ (with 0.1 s^−1^) may decrease the time for dynamic spheroidization in stage II (with 0.001 s^−1^). Similarly, the raised ${\dot{\varepsilon }}_{2}$ also suppresses the spheroidization process (Figure 3e,f), the volume fraction of un-spheroidized αL phase is 4.11% and the spheroidization fraction is only 39.91% at 1003–0.1 s^−1^–0.3–1 s^−1^ (Figure 3f). This is attributed to the insufficient deformation time for the spheroidization in stage II.

#### 3.2.3. DRX Behavior of β Phase

**Figure 4:**
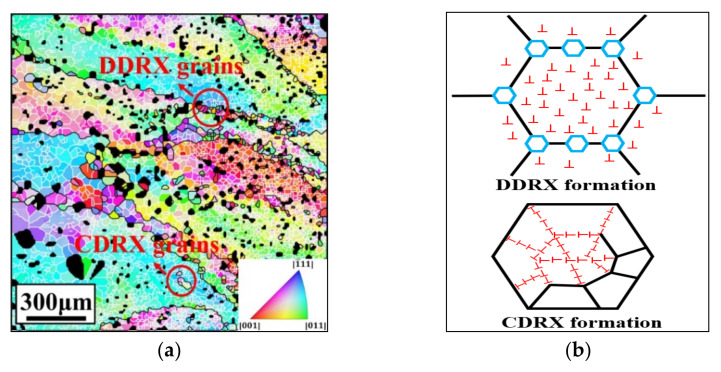
(**a**) IPF of Ti-55511 alloy compressed at 1003 K–0.1 s^−1^–0.3–0.001 s^−1^ (Note: Black lines and white lines indicate HAGBs and LAGBs, respectively, α phases are filled in black.); (**b**) Schematic diagram of β DRX formations.

As previously reported [1,6], DRX is the main deformation characteristic of the β phase in the hot compressed Ti-55511 alloy. Generally, the DRX mechanism can divided into CDRX and DDRX [42,43]. CDRX is related to the formation of subgrains while DDRX is related to the nucleation-growth of strain-free new grains [44,45]. Figure 4a shows the inverse pole figures (IPF) at 1003 K–0.1 s^−1^–0.3–0.001 s^−1^, in which the black and white lines indicate HAGBs (>15°) and LAGBs (2–15°), respectively. Many new small grains with HAGBs appear on the boundaries of the deformed original β phases, which are induced by DDRX. In this process, new strain-free grains nucleate at high-density regions and grow by consuming the neighboring dislocations. Meanwhile, many LAGBs and few HAGBs can be observed in original β phases. These are the typical characteristics of CDRX. The dislocations are rearranged and sub-grain boundaries (LAGBs) appear. Then, the misorientation of LAGBs increases by the rotation of sub-grains and the consuming of dislocations during deformation [46,47,48]. Eventually, these LAGBs transform into HAGBs. The visualized schematic diagram of CDRX and DDRX is shown in Figure 4b.

Based on the measured EBSD data, the β sub-grain size and DRX fraction are calculated by METX5.6.0. The DRX grains are identified based on the grain orientation spread (GOS), which is the average misorientation angle of grain reference orientation deviations of each grain. The computing method is as follows [49]:

(2) $$GOS=\frac{1}{J(i)}\sum_{j}\omega_{ij}$$

where J(i) is the number of pixels in grain i, $\omega_{ij}$ is the mis-orientation angle between the orientation of pixel j and mean orientation of grain i. Here, the grains with GOS < 3° are identified as DRX grains. The dynamic recrystallization fraction and average sub-grain size of β phases are shown in Table 3.

**Figure 5:**
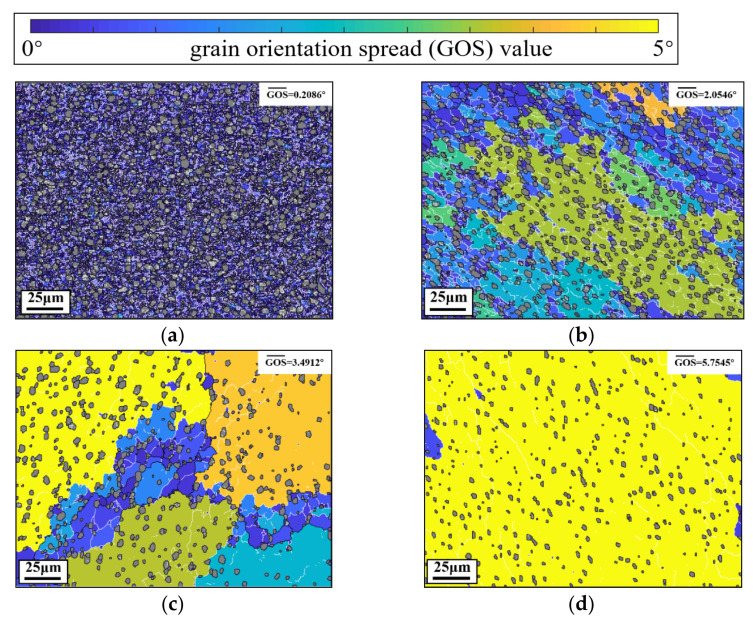
GOS maps of the Ti-55511 alloy compressed at different temperatures: (**a**) 973 K–0.1 s^−1^–0.3–0.001 s^−1^; (**b**) 1003 K–0.1 s^−1^–0.3–0.001 s^−1^; (**c**) 1033 K–0.1 s^−1^–0.3–0.001 s^−1^; (**d**) 1063 K–0.1 s^−1^–0.3–0.001 s^−1^.

Obviously, the sub-grain size increases with the increased T and decreased ${\dot{\varepsilon }}_{2}$. It indicates that the high temperature and low strain rate promote the growth of β subgrains. However, the influence of deformation conditions on the DRX degree of β phase is not clear. Figure 5 depicts the GOS maps of the compressed alloy at different temperatures.

**Figure 6:**
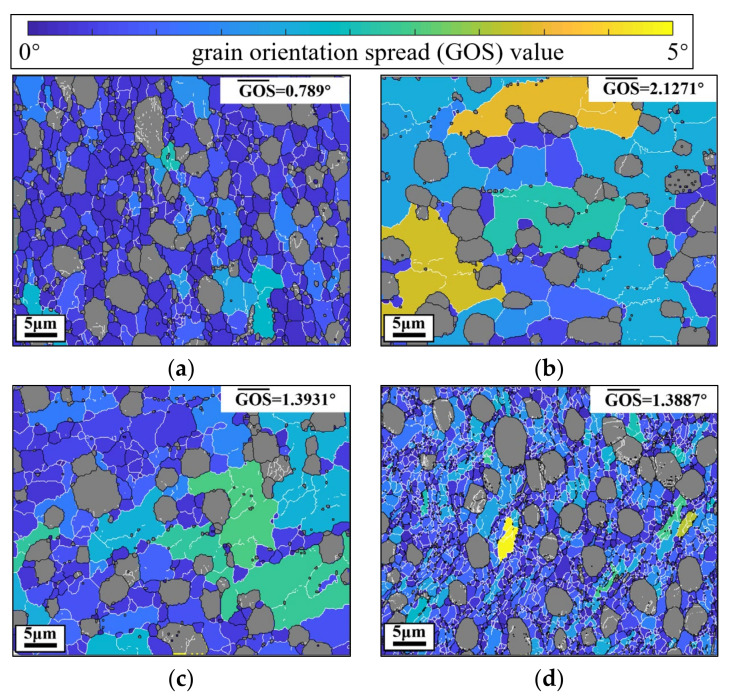
GOS maps of the Ti-55511 alloy compressed at: (**a**) 1003 K–0.1 s^−1^–0.5–0.001 s^−1^; (**b**) 1003 K–0.1 s^−1^–0.3–0.001 s^−1^; (**c**) 1003 K–0.1 s^−1^–0.3–0.01 s^−1^; (**d**) 1003 K–0.1 s^−1^–0.3–1 s^−1^.

Here, α phases are filled in gray to highlight β phases. In Figure 5a, the mean GOS is low (0.2086°) while the DRX fraction is high (99.07%) at 973 K–0.1 s^−1^–0.3–0.001 s^−1^. Many LAGBs exist in the β matrix, and these LAGBs result from the rearrangement of dislocations in CDRX process [6,17]. Thus, β subgrains with LAGBs are CDRX grains, which indicates that CDRX is the dominant DRX mechanism of β phase. Meanwhile, subgrains are refined (about 1.69 μm), because the high α phase content (54%) at 973 K can impede the growth of β subgrains. As indicated in Figure 5b, when the temperature is raised from 973 K to 1003 K, the mean β subgrains size significantly increases from 1.69 μm to 9.73 μm. Some DRX grains with HAGBs appear on the grain boundaries of original β grains. Meanwhile, there are many LAGBs and few HAGBs distributed within original β grains, which is the characteristic of the CDRX process [9]. When the temperature is increased to 1033 K, some DRX grains appear on the grain boundary triple junctions. Almost no subgrains exist in the original β interior. The length of LAGBs is apparently higher than those at 1003 K and 973 K. Therefore, it can be concluded that DDRX is the main DRX mechanism of the Ti-55511 alloy when the T is higher than 1033 K. At 1063 K (Figure 5d), few subgrains can be observed in the 220 μm × 160 μm regions. The β subgrains are large (17.08 μm), while the DRX fraction is low (1.88%). This is because the high T suppresses the CDRX and promotes the growth of the grains. Figure 6a,b presents the GOS maps at different $\varepsilon_{I}$.

Obviously, the mean GOS decreases when the $\varepsilon_{I}$ is increased from 0.2 to 0.5. This is because the larger $\varepsilon_{I}$ (with 0.1 s^−1^) means less time for dislocation annihilation in stage II (with 0.001 s^−1^), and the high substructures density and CDRX degree are obtained. Meanwhile, it means less time for the dissolving of α phases, and the higher content of α phases can impede the growth of β subgrains. Similarly, when the ${\dot{\varepsilon }}_{2}$ is increased from 0.001 s^−1^ to 1 s^−1^, the mean GOS decreases from 2.1271° to 1.3887°. Meanwhile, the β subgrains size decreases from 9.73 μm to 1.48 μm. In general, the experimental results manifest that the low temperature/high strain rate can promote CDRX and decrease the subgrains size of β phases, while the high temperature/low strain rate can promote DDRX nucleation/HAGBs movement/α phases dissolution, accelerating the growth of β grains/subgrains [17,45].

## 4. Development of a Unified Physical-Based Model

Material plastic behavior is usually described by constitutive equations. In the following sections, a set of constitutive equations incorporating dislocation interaction, phases transformation, DRX of β phase and spheroidization of α phase are developed to describe the flow behavior and microstructure evolution of the studied Ti alloy.

### 4.1. Constitutive Law

Normally, the total flow stress consists of two main contributions according to thermal activation theory [50], as shown in Equation (3). However, the effect of grain boundary (Hall–Petch effect) is negligible for typical titanium alloys [45].

(3) $$\sigma =\sigma_{ath}+\sigma_{th}$$

where $\sigma_{ath}$ is the athermal stress related to the dislocation forest [51], $\sigma_{th}$ is the thermally activated stress.

**Figure 7:**
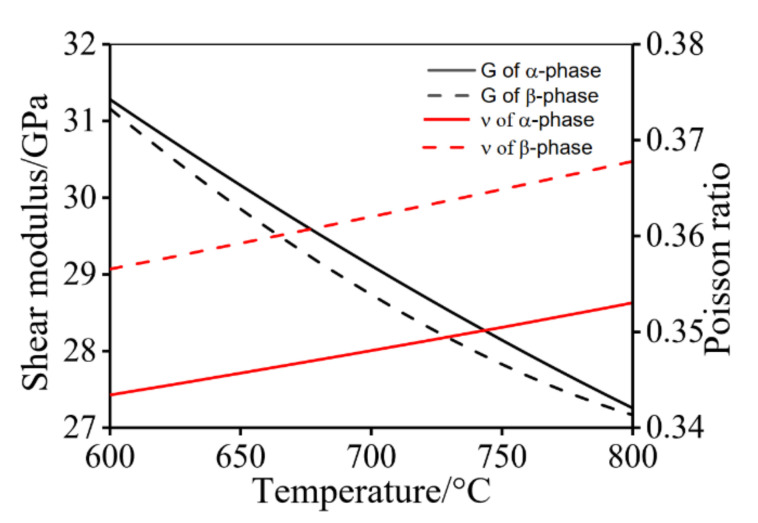
Calculated data using JMatProV7.0: shear modulus and Poisson ratio of α and β phases.

$\sigma_{ath}$ is associated with the dislocation density, i.e.,

(4) $$\sigma_{ath}=M\alpha \mu b\sqrt{\rho }$$

where *M* is Taylor constant factor (3.06), α is proportional constant (0.5), *μ* is shear modulus and calculated by JMatProV7.0 at different temperature (Figure 7). *b* is the Burgers vector (2.95 × 10^−10^ m^−1^ for α phase and 2.86 × 10^−10^ m^−1^ for β phase), and ρ represents mean dislocation density (including mobile and immobile dislocation).

$\sigma_{th}$ is calculated by Arrhenius-type equation [45]:

(5) $$\sigma_{th}=\frac{1}{\alpha_{th}}\ln\{{\left(\frac{\dot{\varepsilon }\exp(\frac{Q_{th}}{RT})}{A_{th}}\right)}^{\frac{1}{n_{th}}}+{\left[{\left(\frac{\dot{\varepsilon }\exp(\frac{Q_{th}}{RT})}{A_{th}}\right)}^{\frac{1}{2n_{th}}}+1\right]}^{0.5}\}$$

where $\alpha_{th}$, $n_{th}$, $A_{th}$ and $Q_{th}$ are material constants, *R* is the gas universal constant (8.314 KJ/mol).

### 4.2. Microstructure Models 

#### 4.2.1. Modelling the Evolution of Dislocation Density

During the metal plastic deformation, the dislocation density is usually affected by two aspects: one is the dislocations accumulation via WH, and the other is the rearrangement and annihilation of dislocations via DRV. According to the Kocks–Mecking (K–M) model [50], the change of ρ can be described as:

(6) $$\dot{\rho }={\dot{\rho }}_{WH}^{+}-{\dot{\rho }}_{DRV}^{-}$$

Generally, the increment rate of ρ is inversely proportional to the dislocation mean free path (Λ) and proportional to strain rate ($\dot{\varepsilon }$). So the increment rate of dislocation density is assumed as [31]:

(7) $${\dot{\rho }}_{WH}^{+}=\frac{M}{b}\frac{1}{\Lambda }\dot{\varepsilon }$$

For titanium alloys, the Λ is close to the dislocation substructures and grain boundaries of α/β phases [45]. Therefore, the Λ is assumed as the total of the inverse of average β subgrains size, equiaxed α size and lamellar α thickness:

(8) $$\frac{1}{\Lambda }=(f_{\beta }\frac{1}{s}+f_{\alpha_{E}}\frac{1}{d_{E}}+f_{\alpha_{L}}\frac{1}{w_{L}})$$

where $f_{\beta }$, $f_{\alpha_{E}}$ and $f_{\alpha_{L}}$ are the fraction of different phases, which can be calculated by Equation (27); $d_{E}$ is the initial equiaxed α size (3.1 μm); $w_{L}$ is the initial thickness of lamellar α phase (0.245 μm); s is the diameter of substructures in β matrix.

The mean size of β subgrains is related to the dislocation cell/substructure [45], i.e.,

(9) $$s_{\beta }=\kappa \frac{k_{w}}{\sqrt{\rho }}$$

where κ is the coefficient of substructures and β subgrains, $k_{w}$ is the WH coefficient related to $\dot{\varepsilon }$ and temperature (*T*) [52]:

(10) $$k_{w}=A_{w}{\left[\dot{\varepsilon }\exp(\frac{-Q_{w}}{RT})\right]}^{n_{w}}$$

where $A_{w}$, $Q_{w}$ and $n_{w}$ are material constants.

The dislocation annihilation caused by DRV can expressed as:

(11) $${\dot{\rho }}_{DRV}^{-}=k_{v}\rho \dot{\varepsilon }$$

where $k_{v}$ is related to $\dot{\varepsilon }$ and *T*:

(12) $$k_{v}=A_{v}{\left[\dot{\varepsilon }\exp(\frac{-Q_{v}}{RT})\right]}^{n_{v}}$$

where $A_{v}$, $Q_{v}$ and $n_{v}$ are material constants.

However, for titanium alloys, the DRX of β phase and spheroidization of lamellar α phase would consume the dislocation structure during deformation, leading to the dynamic softening [3,6]. Therefore, in this work, two additional softening components (${\dot{\rho }}_{DRX}^{-}$ and ${\dot{\rho }}_{SPH}^{-}$) are introduced to depict the effects of DRX and spheroidization on ρ. So Equation (6) can be updated as:

(13) $$\dot{\rho }={\dot{\rho }}_{WH}^{+}-{\dot{\rho }}_{DRV}^{-}-f_{\beta }{\dot{\rho }}_{DRX}^{-}-f_{\alpha_{L}}{\dot{\rho }}_{SPH}^{-}$$

Considering the DRX of β phase and the spheroidization of α_L_ phase, the evolution of ρ can be modified as,

(14) $$\dot{\rho }=(\frac{M}{b}\frac{1}{\Lambda })\dot{\varepsilon }-k_{v}\rho \dot{\varepsilon }-f_{\alpha_{L}}\frac{k_{s}\rho \dot{S}}{1-S}-f_{\beta }\frac{k_{x}(\rho -\rho_{0})}{{\left(1-X\right)}^{\phi }}\dot{X}$$

where the ‘dot’ represents the change rate of time, S is the spheroidization fraction of lamellar α phase, X is the DRX fraction of β phase, $\rho_{0}$ is the initial dislocation density (10^12^ m^−2^), $k_{s}$, $k_{x}$ and ϕ are material constants.

#### 4.2.2. Modelling the DRX Softening

As shown in Figure 6, both CDRX and DDRX occur in the β matrix during hot deformation. Here, the process including the nucleation and growth of new free-stress grains (DDRX) is considered. The change rate of DRX fraction of β phase can be expressed as [53]:

(15) $$\dot{X}=k_{n}\dot{N}{\left(1-X\right)}^{c_{0}}v$$

where $k_{n}$ and $c_{0}$ are material constants, v is the velocity of the recrystallized region sweeps through the un-recrystallized region, $\dot{N}$ is the nucleation rate of DDRX grains.

(16) $$\dot{N}=N_{i}(\frac{\rho }{\rho_{c}}){\dot{\varepsilon }}^{c_{1}}\exp(-\frac{Q_{N}}{RT})$$

where $N_{i}$, $c_{1}$, $Q_{N}$ are material constants, $\rho_{c}$ calculated in Equation (21) is critical dislocation density to start DRX process.

The v is related to the boundary migration, i.e.,

(17) $$v=M_{bm}P$$

where $M_{bm}$ is the GB mobility, P is the drive force, which can be calculated by [17]:

(18) $$P=\frac{\mu b^{2}}{2}\rho$$

$M_{bm}$ can be calculated by [31]:

(19) $$M_{bm}=\frac{b\delta D_{ob}}{k_{b}T}\exp(-\frac{Q_{m}}{RT})$$

where δ is the GB thickness; $D_{ob}$ and $Q_{m}$ are the self-diffusion coefficient and activation energy, respectively; $k_{b}$ is the Boltzmann constant (1.38 × 10^−23^ J/K).

Combining Equations (15)–(19), the change rate of X can be expressed as:

(20) $$\dot{X}=k_{n}\frac{b\delta D_{ob}}{k_{b}T}\exp(-\frac{Q_{m}}{RT})N_{i}(\frac{\rho }{\rho_{c}}){\dot{\varepsilon }}^{c_{1}}\exp(-\frac{Q_{N}}{RT}){\left(1-X\right)}^{c_{1}}$$

When the ρ reaches a critical value, the new grains are generated and DDRX takes place. Once the critical dislocation density ($\rho_{c}$) appears, the critical strain ($\varepsilon_{c}$) can be acquired. Normally, the $\rho_{c}$ to start DRX can be expressed as [54]:

(21) $$\rho_{c}={\left(\frac{20\gamma \dot{\varepsilon }}{3bLM_{bm}\lambda^{2}}\right)}^{\frac{1}{3}}$$

where γ is the grain boundary energy, which is related to the grain boundary misorientation; λ is the dislocation line energy given by $\lambda =\frac{1}{2}\mu b^{2}$; *L* is the initial dislocation mean free path. For the HAGBs, γ is calculated by [31]:

(22) $$\gamma =\frac{\mu b\theta_{m}}{4\pi (1-\upsilon )}$$

where $\theta_{m}$ represents the critical misorientation of HAGBs (15o), υ is the Poisson ratio calculated by JMatProV7.0 in Figure 4. L is given by [31]:

(23) $$L=kb{\left(\frac{\mu }{\sigma_{th}}\right)}^{m}$$

where k and m are material parameters.

#### 4.2.3. Modelling the Spheroidization Softening

As shown in Figure 4, many lamellar α phases are broken in spheroidized α phases during hot deformation. Semiatin et al. [55] verified that the spheroidization of lamellar α phases can lower the resistance of dislocation movement. Wang et al. [9] found that spheroidization is a process related to the formation/ movement of GBs. Therefore, the dynamic spheroidization rate can be given as [17]:

(24) $$\dot{S}=\frac{c_{2}(1-S){\dot{\varepsilon }}^{c_{3}}M_{bm}P}{w_{L}}$$

where $c_{2}$ and $c_{3}$ are material constants. 

#### 4.2.4. Modelling the Phase Transformation

As shown in Table 2, the volume fractions of β, α_E_ and α_L_ phases are related to the temperature and time; $f_{\beta }$ increases with the increased temperature and time when the T is higher than 700 °C. The time includes the heating time and deformation time, i.e.,

(25) $$t=t_{heat}+t_{deform}$$

where $t_{heat}$ is the heating time before deformation (300 s in all cases); $t_{deform}$ is deformation time.

(26) $$t_{deform}=\frac{\varepsilon_{I}}{{\dot{\varepsilon }}_{1}}+\frac{\varepsilon_{II}}{{\dot{\varepsilon }}_{2}}$$

where $\varepsilon_{I}$ and $\varepsilon_{II}$ are the true strains of stages I and II, respectively; ${\dot{\varepsilon }}_{1}$ and ${\dot{\varepsilon }}_{2}$ are strain rates of stages I and II, respectively.

The volume fraction of β, α_E_ and α_L_ phases can be expressed by modifying the JMAK equations [56]:

(27) $$\left\{\begin{array}{l} f_{\beta }=1-\varphi_{1}(\frac{t}{t_{ref}}{)}^{\varphi_{2}}(\exp(\varphi_{3}(\frac{T_{\beta }-T}{T_{\beta }})){)}^{\varphi_{4}}\\ f_{\alpha_{L}}=\varphi_{5}(\frac{t}{t_{ref}}{)}^{\varphi_{6}}(\exp(\varphi_{7}(\frac{T_{\alpha_{E}}-T}{T_{\alpha_{E}}})){)}^{\varphi_{8}}\\ f_{\alpha_{E}}=1-f_{\beta }-f_{\alpha_{L}}\\ f_{\alpha_{SP}}=S\times f_{\alpha_{L}} \end{array}\right.$$

where $\varphi_{1}$, $\varphi_{2}$, $\varphi_{3}$, $\varphi_{4}$, $\varphi_{5}$, $\varphi_{6}$, $\varphi_{7}$ and $\varphi_{8}$ are material constants; $T_{\beta }$ represents the β-transformation temperature (1148 K); $T_{\alpha_{E}}$ represents the temperature which all lamellar α phases are dissolved (1093 K) [57]. The $t_{ref}$ is 1022 s. The temperature is constant during deformation.

### 4.3. Determination of Material Constants

Due to the high coupled nonlinear and differential of equations in the developed unified model, a GA-based algorithm is used to optimize the material constants via minimizing the residuals of experimental and calculated values. The parameters and corresponding procedure of the GA-based algorithm are described elsewhere [25,26,58]. 

The material constants are determined in two steps. Firstly, a group of initial values and the domains of material parameters are determined according to their physical significance. Secondly, the experimental data are used to optimize the material parameters. Then, the $\varepsilon_{c}$ for start DRX ($\varepsilon_{c}$) is determined by $\rho_{c}$. When $\varepsilon <\varepsilon_{c}$, the change rate of DRX ($\dot{X}$) is set to 0. In this work, four sub-objective functions are defined:

(28) $$\left\{\begin{array}{l} f_{1}(x)=\sum_{i}w_{i}((\sigma_{i}^{c}-\sigma_{i}^{e})/\sigma_{i}^{e}{)}^{2}\\ f_{2}(x)=\sum_{j}w_{j}((X_{j}^{c}-X_{j}^{e})/X_{j}^{e}{)}^{2}\\ f_{3}(x)=\sum_{k}w_{k}((S_{k}^{c}-S_{k}^{e})/S_{k}^{e}{)}^{2}\\ f_{4}(x)=\sum_{k}w_{l}((s_{\beta l}^{c}-s_{\beta l}^{e})/s_{\beta l}^{e}{)}^{2} \end{array}\right.$$

where $f_{1}(x)$, $f_{2}(x)$, $f_{3}(x)$ and $f_{4}(x)$ are the residuals for experimental results and calculated results; $x=[x_{1},x_{2},\ldots x_{s}]$ stands for material constants; $\sigma_{i}^{c}$ and $\sigma_{i}^{e}$ are the computational and experimental stresses, respectively; $X_{j}^{c}$ and $X_{j}^{e}$ are the computational and experimental DRX fractions of β phase, respectively; $S_{k}^{c}$ and $S_{k}^{e}$ are the computational and experimental spheroidization fractions of α_L_ phase, $s_{\beta l}^{c}$ and $s_{\beta l}^{e}$ are the computational and experimental average grain sizes of β subgrains; $w_{i}$, $w_{j}$, $w_{k}$, $w_{l}$ are the weighting factors and given as 0.25, 0.25, 0.25 and 0.25, respectively. The global objective function is the sum of four sub-objective functions:

(29) $$f(x)=f_{1}(x)+f_{2}(x)+f_{3}(x)+f_{4}(x)$$

The determined material parameters are listed in Table 4.

## 5. Results and Discussion

### 5.1. Validation of the Unified Constitutive Model

**Figure 8:**
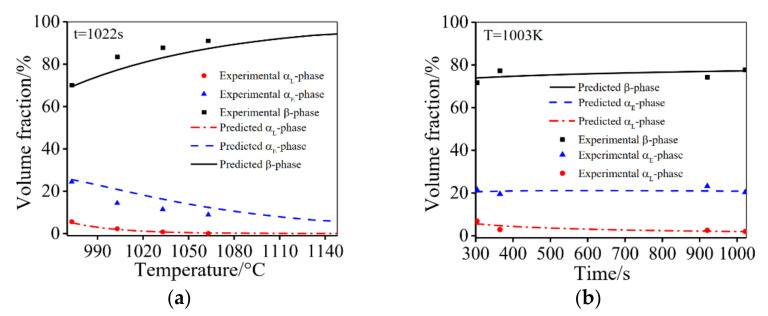
The volume fractions of α_E_, α_L_ and β phases at different: (**a**) temperatures; (**b**) times.

Figure 8 shows a comparison of the computed (solid curve) and experimental (symbol) fractions of α_E_, α_L_ and β phases at different temperatures and times.

**Figure 9:**
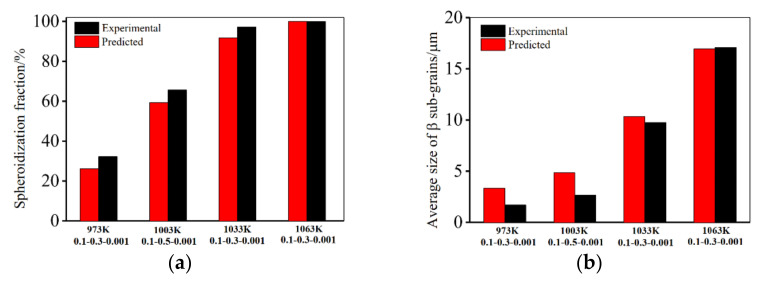
Comparisons of the calculated and experimental: (**a**) spheroidization fractions of α_L_; (**b**) average sizes of β subgrains.

It is evident that there is a good agreement between the computed and experimental results. This indicates that the established model (Equation (27)) is effective. Figure 9 shows the experimental and predicted material parameters under different deformation conditions. A good consistency between the calculated and experimental material parameters is achieved. The evolution law of phase transformation, β subgrains, spheroidization fractions under different deformation condition are also consistent with the experimental results in Section 3.2.

**Figure 10:**
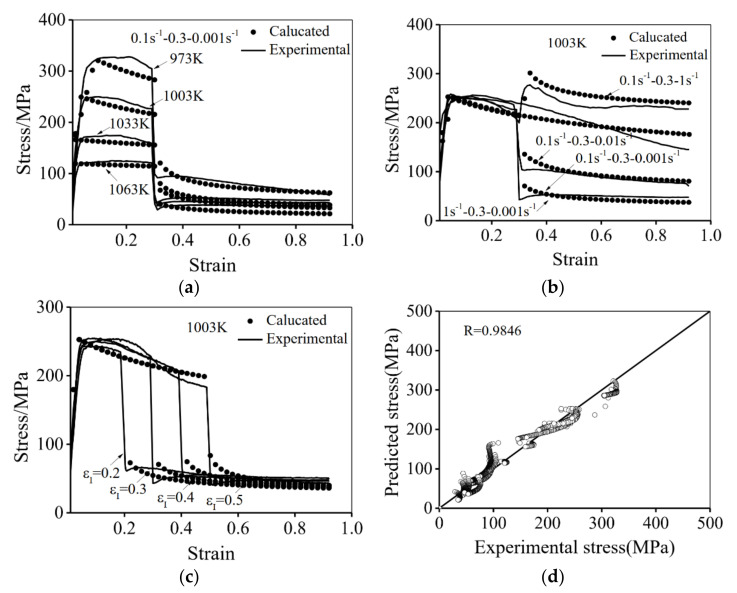
Comparisons of the calculated (solid line) and experimental (symbol) strain-stress curves at different: (**a**) temperatures; (**b**) ${\dot{\varepsilon }}_{2}$; (**c**) $\varepsilon_{I}$; (**d**) correlation coefficient.

Figure 10 shows the experimental (solid line) and calculated (symbol) flow stress curves under different deformation conditions. A good prediction accuracy is obtained. The correlation coefficient (R) is calculated by:

(30) $$R=\frac{\sum_{i=1}^{N}\left(E_{i}-\bar{E})(P_{i}-\bar{P}\right)}{\sqrt{\sum_{i=1}^{N}{\left(E_{i}-\bar{E}\right)}^{2}{\left(P_{i}-\bar{P}\right)}^{2}}}$$

where $E_{i}$ is the experimental result, $P_{i}$ is the predicted result, $\bar{E}$ is the average experimental stresses, $\bar{P}$ is the average predicted stresses. As shown in Figure 10d, the R is 0.9846. Therefore, the established unified model is suitable for describing the hot deformation behavior of the Ti-55511 alloy compressed at stepped strain rates.

### 5.2. Prediction of Microstructure Evolution

**Figure 11:**
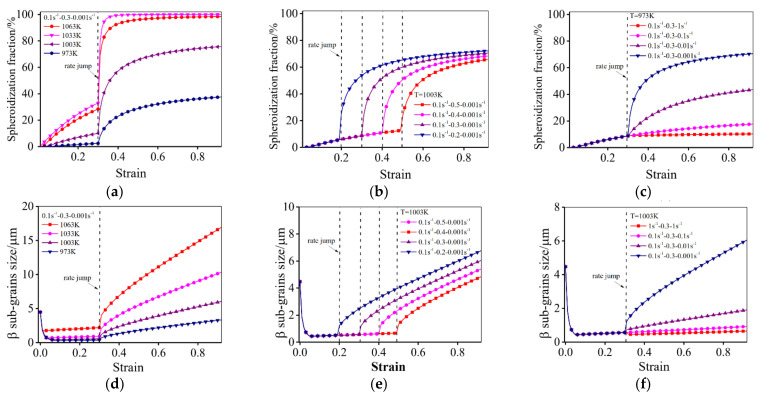

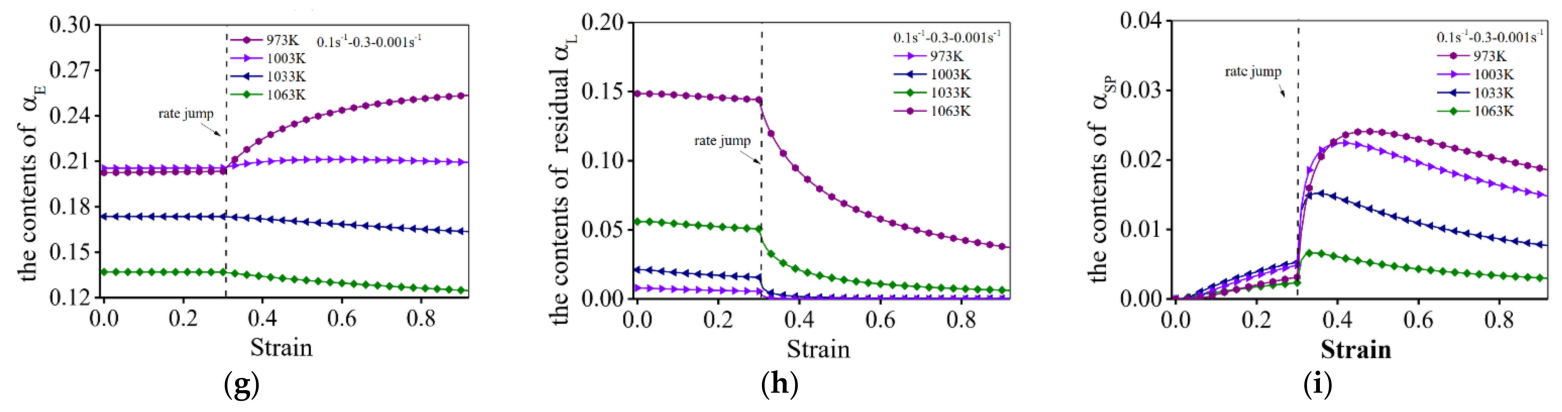
Prediction of microstructure evolution under different testing conditions: (**a**–**c**) spheroidization fraction of α_L_; (**d**–**f**) β subgrains size; (**g**–**i**) the contents of α_E_, α_L_ and α_SP_ phases.

The effects of deformation parameters on the predicted spheroidization fractions of α_L_ phase, β sub-grain size, and the contents of α_E_/α_L_/α_SP_ phases are presented in Figure 11.

It can be seen that the spheroidization velocity of the α_L_ phase increases with increasing the temperature and decreasing the strain rate (Figure 11a–c). This is because a high temperature provides a great driving force for the spheroidization, and a low strain rate means more time for the spheroidization. When the strain rate is changed from 0.1 s^−1^ to 0.001 s^−1^ at the $\varepsilon_{I}$ range of 0.2–0.5, a high

ρ

density generated in stage I can provide a strong driving force for the spheroidization (Equation (24)). Subsequently, the spheroidization in stage II is promoted. In contrast, when the strain rate is changed from 0.001 s^−1^ to 1 s^−1^, the increment rate of spheroidization is lowered. However, the spheroidization continues to proceed at a different rate once the strain rate is changed at $\varepsilon_{I}$.

The predicted mean β sub-grain size under different conditions is shown in Figure 11d–f. For a near β titanium alloy deformed in α+β region, the β sub-grain size is related to the boundary density of dislocations cell/wall [6,7]. When the strain rate is changed from a high value (0.1 s^−1^, 1 s^−1^) to a low value (0.001 s^−1^) at $\varepsilon_{I}$, the β sub-grain size first decreases and then increases at 973–1063 K. This is because the formation of dislocations cells/walls restricts the growth of subgrains. Meanwhile, the size of sub-grain increases due to the reorganization/annihilation of dislocations as well as the formation/movement of HAGBs [6]. In addition, the higher strain rates and lower temperatures can refine the β matrix. This indicates that the generation of dislocations cells/walls is faster than the formation/movement of HAGBs. 

Figure 11g–i shows the variation of the phase contents of α_E_, α_L_ and α_SP_ phases during hot deformation. In Figure 11g–i, the content of the α_E_ phase decreases with the increased strain when deformed at 1033–1063 K, but increases at 973–1003 K (Figure 11h). This indicates that the growth of the α_E_ phase is dominant when deformed at low temperatures. In addition, the content of the α_L_ phase decreases gradually with the increased strain at 973–1063 K, because more time is needed for a phase equilibrium when the temperature is lower [56,59,60]. But the content of the α_SP_ phase first increases to a peak value, then decreases gradually to the total strain. This is ascribed to the spheroidization of the α_L_ phase and the dissolution of the α_SP_ phase.

### 5.3. Computed Results of Multistep Strain Rates Condition

**Figure 12:**
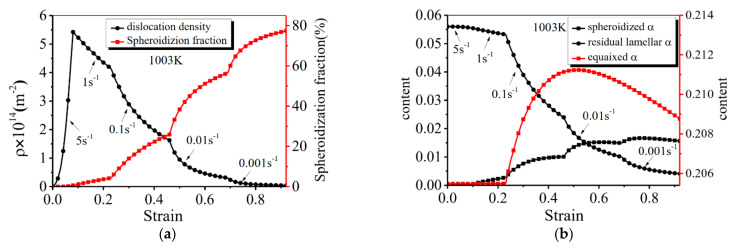
Prediction of microstructure evolution at 5 s^−1^–1 s^−1^–0.1 s^−1^–0.01 s^−1^–0.001 s^−1^: (**a**) dislocation density and spheroidization fraction; (**b**) the contents of the α_E_, α_L_ and α_SP_ phases.

Figure 12 shows the effects of multistep strain rates on the calculated dislocation density, spheroidization fraction of the α_L_ phase, and the contents of the α_E_/α_L_/α_SP_ phases.

In Figure 12a, when the multistep strain rate is 5 s^−1^–1 s^−1^–0.1 s^−1^–0.01 s^−1^–0.001 s^−1^ and the temperature is 1003 K, the *ρ* rapidly increases at the early deformation stage with a high strain rate (5 s^−1^). In contrast, the dislocation density decreases gradually after the strain rate is changed from 5 s^−1^ to 1 s^−1^, and the decreasing rate drops with the decreased strain rate. Meanwhile, the spheroidization fraction of the α_L_ phase increases gradually with the strain, and the increasing rate rises with the decreased strain rate. In Figure 12b, the change of the α_E_, α_L_ and α_SP_ phases is also sensitive to the stepped strain rates. Undoubtedly, the microstructure evolution in the Ti-55511 alloy is significantly affected by the deformation history, which leads to the changed flow stress at a strain rate abrupt saltation [61,62]. Based on the above analysis and discussions, it is concluded that the flow behavior and microstructure evolution can be accurately predicted by the developed unified model.

## 6. Conclusions

The plastic flow behavior and microstructure evolution of a Ti-55511 alloy are researched by hot compression experiments with time-varying strain rates. The phase transformation of the α/β phases, the spheroidization of the α_L_ phase and the DRX of the β phase are quantified and analyzed. The DRX mechanisms of the β phase are also discussed. The constitutive models are established to predict the flow behavior and microstructure evolution. Some significant discoveries and conclusions are listed as follows:

1. The contents of α_L_, α_E_, α_SP_ and β phases, as well as the spheroidization of the α_L_ phase, are significantly influenced by deformation parameters. Increasing the temperature and deformation time can promote the spheroidization of the α_L_ phase and the α→β transformation;
2. Both DDRX and CDRX of the β phase can be observed during the hot compression with stepped strain rates. DDRX mainly occurs on the boundaries of the deformed β phase, while CDRX mainly occurs within the deformed β phase. The β subgrains formed in the CDRX process can refine the microstructure. The low deformation temperature/high strain rate can increase substructural density and thus promote the CDRX process. Meanwhile, CDRX is the main DRX mechanism of the β matrix at the temperature range of 973–1003 K, while DDRX is the dominant DRX mechanism of the β matrix when the temperature is higher than 1033 K;
3. WH behavior is affected by the substructural diameter of β subgrains, the mean diameter of the α_E_ phase and the thickness of the α_L_ phase. In the developed unified model, WH, DRV, the DDRX of β phase and the spheroidization of the α_L_ phase are considered. It can precisely forecast the flow stress and microstructure change of the studied Ti–55511 alloy at stepped strain rates;
4. The dislocation density, spheroidization fractions of the α_L_ phase, and the contents of the α_E_/α_L_/α_SP_ phases are significantly affected by the deformation history, which leads to the variation of the flow stress at strain rate abrupt saltation.

## Figures and Tables

**Table 1:** Schemes of the hot compression tests.

Case ID	Temperature (*T*, K)	Stage I		Stage II	
		Strain Rate ($\varepsilon_{1}$, s^−1^)	True Strain ($\varepsilon_{1}$)	Strain Rate (${\dot{\varepsilon }}_{2}$, s^−^)	True Strain ( $\varepsilon_{II}$)
973 K–0.1 s^−1^–0.3–0.00 1 s^−1^	973	0.1	0.3	0.001	0.62
1003 K–0.1 s^−1^–0.3–0.001 s^−1^	1003	0.1	0.3	0.001	0.62
1033 K–0.1 s^−1^–0.3–0.001 s^−1^	1033	0.1	0.3	0.001	0.62
1063 K–0.1 s^−1^–0.3–0.001 s^−1^	1063	0.1	0.3	0.001	0.62
1003 K–0.1 s^−1^–0.2–0.001 s^−1^	1003	0.1	0.2	0.001	0.72
1003 K–0.1 s^−1^–0.4–0.001 s^−1^	1003	0.1	0.4	0.001	0.52
1003 K–0.1 s^−1^–0.5–0.001 s^−1^	1003	0.1	0.5	0.001	0.42
1003 K–0.1 s^−1^–0.3–0.01 s^−1^	1003	0.1	0.3	0.01	0.62
1003 K–0.1 s^−1^–0.3–1 s^−1^	1003	0.1	0.3	1	0.62

**Table 2:** The volumes fraction of α_E_, α_L_, α_SP_, β phases and spheroidization fraction (%).

Case ID	α_L_ Phase (%)	α_E_ Phase (%)	α_SP_ Phase (%)	β Phase (%)	Spheroidized Fraction (%)
973 K–0.1 s^−1^–0.3–0.001 s^−1^	5.61	24.4	1.47	69.99	26.15
1003 K–0.1 s^−1^–0.3–0.001 s^−1^	0.736	11.43	0.73	87.73	91.75
1063 K–0.1 s^−1^–0.3–0.001 s^−1^	0.007	8.9	0.007	91.03	100
1003 K–0.1 s^−1^–0.2–0.001 s^−1^	2	20.39	1.67	77.65	84.91
1003 K–0.1 s^−1^–0.3–0.001 s^−1^	2.22	14.38	1.78	83.39	80.12
1003 K–0.1 s^−1^–0.5–0.001 s^−1^	1.39	14.74	0.82	83.87	59.31
1003 K–0.1 s^−1^–0.3–0.01 s^−1^	2.89	19.54	2.57	77.24	79.72
1003 K–0.1 s^−1^–0.3–1 s^−1^	6.84	21.53	2.73	71.63	39.92

**Table 3:** The dynamic recrystallization fraction and average sub-grain size of β phases.

Case ID	Dynamic Recrystallization Fraction (%)	β Sub-Grain Size (μm)	Mean GOS (°)
973 K–0.1 s^−1^–0.3–0.001 s^−1^	99.07	1.69	0.2086
1033 K–0.1 s^−1^–0.3–0.001 s^−1^	31.66	9.76	3.4912
1063 K–0.1 s^−1^–0.3–0.001 s^−1^	1.88	17.08	5.7545
1003 K–0.1 s^−1^–0.3–0.001 s^−1^	79.17	9.73	2.0546
1003 K–0.1 s^−1^–0.5–0.001 s^−1^	99.46	2.66	0.789
1003 K–0.1 s^−1^–0.3–0.01 s^−1^	91.46	4.92	1.3931
1003 K–0.1 s^−1^–0.3–1 s^−1^	94.95	1.48	1.3887

**Table 4:** Determined material parameters in the model.

Material Parameter	Value	Material Parameter	Value
$A_{w}$ $n_{w}$ $Q_{w}(\mathrm{kJ}/\mathrm{mol})$	14.8589	$A_{v}$	0.9037
	0.1016	$n_{v}$	0.0044
	1.3223	$Q_{v}(\mathrm{kJ}/\mathrm{mol})$	247.6467
$k_{n}$ $N_{i}$ $Q_{N}(\mathrm{kJ}/\mathrm{mol})$	85.2429	$k_{x}$	0.6913
	1.0718 × 10^20^	κ	1.8122 × 10^6^
	75.6538	$Q_{m}(\mathrm{kJ}/\mathrm{mol})$	615.1746
$c_{0}$	46.4339	$c_{1}$	0.0945
$c_{2}$	1.1325 × 10^24^	$c_{3}$	0.1155
ϕ	−138.8566	$k_{s}$	31.11
δ	6.946 × 10^−9^	$k_{d}$	1.3404
$n_{th}$	1.6939	$\alpha_{th}$	0.0305
$A_{th}$	6.9472 × 10^7^	$Q_{th}(\mathrm{kJ}/\mathrm{mol})$	197.74
m	11.5903	$\varphi_{1}$	1.4643
$\varphi_{2}$	−0.1128	$\varphi_{3}$	0.0548
$\varphi_{4}$	7.7026	$\varphi_{5}$	5.1559
$\varphi_{6}$	0.001	$\varphi_{7}$	−0.8731
$\varphi_{8}$	6.8973	$D_{ob}$	0.0174

## Data Availability

The raw/processed data required to reproduce these findings cannot be shared at this time as the data also form part of an ongoing study.
